# Leptomeningeal Carcinomatosis in Recurrent Non-Small Cell Lung Cancer: A Case Report and Review of Current Treatment Modalities

**DOI:** 10.7759/cureus.1242

**Published:** 2017-05-12

**Authors:** Saqib Abbasi, Elias Moussaly, Jean P Atallah

**Affiliations:** 1 Internal Medicine, Staten Island University Hospital; 2 Hematology / Oncology, Staten Island University Hospital

**Keywords:** leptomeningeal carcinomatosis, non-small cell lung cancer, egfr mutation, alk, targeted therapy

## Abstract

Leptomeningeal carcinomatosis (LC) is an uncommon sequelae of non-small cell lung cancer. The treatment modalities for LC have historically been limited with an overall poor prognosis. This case report outlines a 76-year-old female who presented with recurrence of non-small cell lung cancer as LC. LC is difficult to treat, and options include radiation, chemotherapy (systemic and intrathecal), as well as targeted therapies. This case outlines a unique approach and reviews the current literature on the effectiveness of these options in non-small cell lung cancer.

## Introduction

Leptomeningeal carcinomatosis (LC) affects five percent of patients with non-small cell lung cancer (NSCLC), and the incidence is increasing. This increased incidence is thought to be secondary to the prolonged survival of cancer patients. Nonetheless, patients with diagnosed LC have a poor prognosis, with an average survival of four to six weeks among untreated patients vs. four to six months among treated patients [1]. In this report, we describe the case of a patient who presented with a recurrent adenocarcinoma of the lung that manifested as LC.

## Case presentation

We present a case of a 76-year-old Caucasian female with a medical history that included hypertension, diabetes, and stage IB (T2a N0 Mx) adenocarcinoma of the lung. At the time of cancer diagnosis, she underwent lobectomy of the affected right upper lobe. The surgical margins were negative on pathology, and she received adjuvant chemotherapy comprising pemetrexed plus carboplatin for three months. Her clinical course was good, and she reported no complaints during regular routine follow-ups. After a 1.5-year follow-up period, she noticed intermittent numbness on the left side of her face, along with insidious, increasingly worsening left-sided hearing loss and weekly headaches that she described as a sharp shooting pain, mostly located over her left scalp, of less than 60 minutes’ duration. Two months after these symptoms started, she began to experience “dizzy spells” and intermittent double vision. Upon continuation of these symptoms, she was subsequently examined at the clinic.

**Figure 1:**
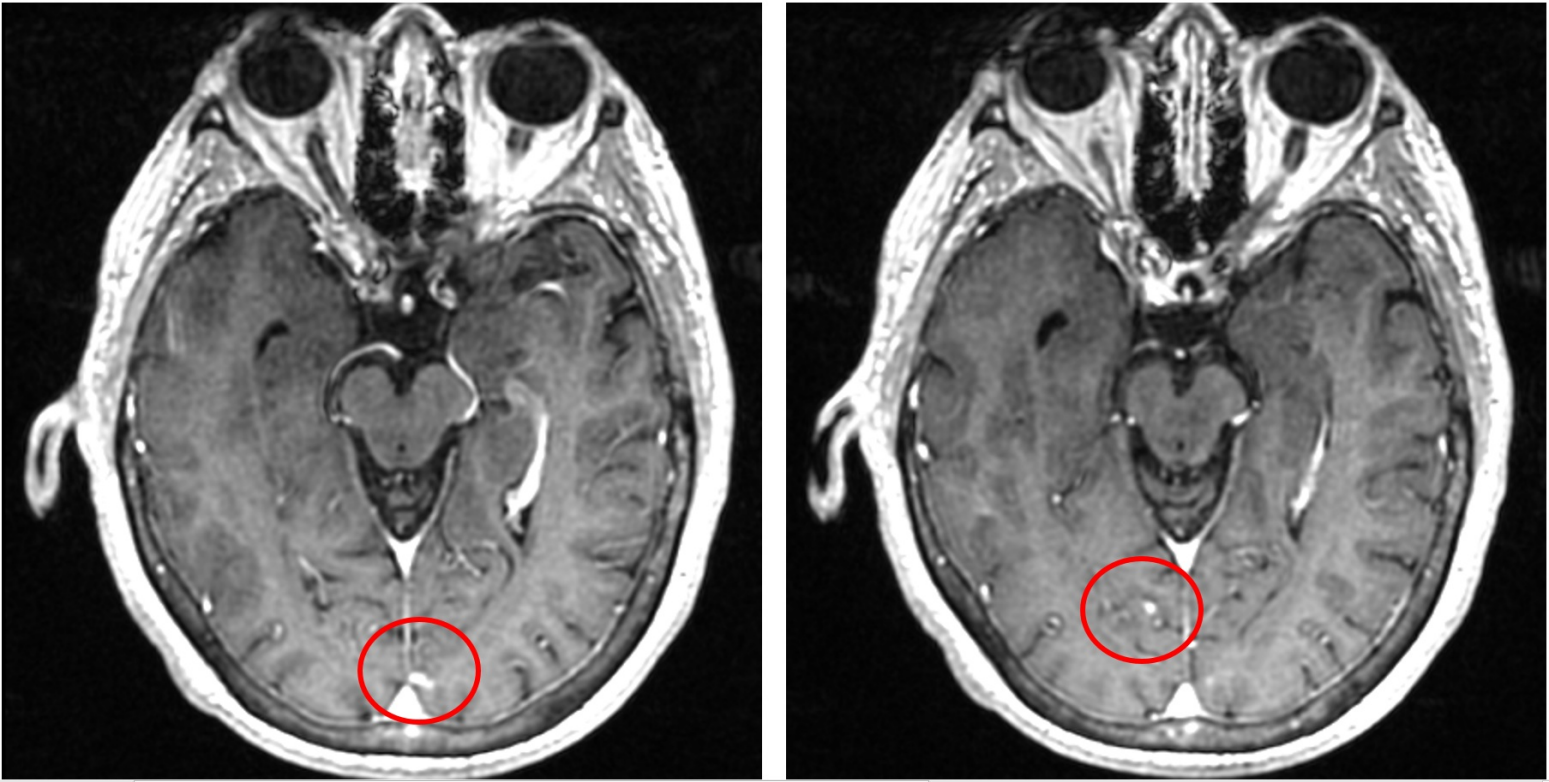
MRI of the brain showing multiple foci of superficial enhancement in the occipital region compatible with metastatic disease (red circles)

At the time of the examination, her Eastern Cooperative Oncology Group performance status was 1. Brain magnetic resonance imaging (MRI) revealed the development of several punctate foci with superficial enhancement bilaterally within the occipital regions, consistent with metastatic disease (leptomeningeal vs. cortical location). No significant mass or edema was observed (Figure 1). Given her history of cancer, blood work, a diagnostic lumbar puncture, and a positron emission tomography (PET)/computed tomography (CT) scan were recommended to address the suspected recurrence. Following a lumbar puncture, her cerebrospinal fluid (CSF) and cytology results were negative (Table 1).

**Table 1:** Cerebrospinal fluid results

Parameter	Value
Red cell count	48 / mm3
White cell count	0 / mm3
Color	Colorless
Appearance	Clear
Glucose	141 mg/dl
Total protein	25.1 mg/dl
Albumin	<0.5 g/dl

However, the PET/CT scan revealed bilateral hilar uptake (6.2 and 5.3), consistent with a recurrence of cancer. No other significant uptake was identified. Combined with the brain MRI findings, we made a diagnosis of the leptomeningeal spread of a recurrent adenocarcinoma of the lung.

The patient received one cycle of pemetrexed and bevacizumab for systemic disease. The tumor was positive for an exon 19 deletion, at which point we initiated a discussion regarding the use of epidermal growth factor receptor (EGFR)-tyrosine kinase inhibitor (TKI) therapy. At three months post initiation of the treatment, the patient was no longer complaining of headaches and double vision. Her left-sided facial numbness and hearing loss markedly improved as well.

## Discussion

Decisions regarding optimal cancer treatment modalities for leptomeningeal carcinomatosis depend on the patient, presentation, risk status, and cancer type. Whole-brain radiation therapy (WBRT), intrathecal chemotherapy (IC), ventriculoperitoneal shunting, EGFR-TKIs and, more recently, anaplastic lymphoma kinase (ALK) inhibitor therapy [2] have yielded favorable outcomes in selected patients.

WBRT is a useful palliative therapy but does not appear to improve survival. In a retrospective study of LC secondary to NSCLC by Morris, et al. (2012), no significant difference in median survival was observed between 46 patients who received WBRT vs. 59 who did not (p = 0.84) [3]. In addition to the transient benefits, WBRT often causes unbearable side effects and carries a significant risk of cognitive decline, especially in patients older than 75 years of age. This modality is therefore often reserved for patients with a poor functional status (Karnofsky performance score <60), encephalopathy, multiple serious neurologic deficits, bulky central nervous system (CNS) disease, and extensive systemic disease with few treatment options.

Although IC has been successfully used to treat LC in patients with lymphoma and breast cancer, no real consensus has been reached regarding IC for lung cancer. Traditionally, methotrexate and cytarabine are not considered active against lung cancers [4-5], and early studies of LC indicate little or no response to these agents [4-5]. Topotecan, which is occasionally administered systemically to patients with NSCLC, does not yield any real benefits when compared to other IC therapies, as demonstrated by Groves, et al. (2008) in a multicenter study of LC secondary to all solid tumors. This finding was attributed to the spread of cancer between the dura and arachnoid, rather than contiguous with the cerebrospinal fluid (CSF). Notable side effects of IC include meningitis, seizures, and dizziness.

Systemic chemotherapy (SC) has yielded mixed results as a result of limited leptomeningeal penetration. Most chemotherapeutic agents do not achieve good CNS penetration. The benefit of SC was primarily attributed to the treatment of systemic disease, rather than any effects on the course of leptomeningeal spread. However, pemetrexed and bevacizumab have been shown to be effective against CNS metastasis. Pemetrexed improves survival, both as an initial treatment option and in refractory cases with CNS involvement, as shown by Beerz, et al. (2010) in a study of 39 patients where 70% of the patients achieved stable disease or a partial response with a median survival of 10 months. Bevacizumab has traditionally been used to treat glioblastoma multiforme and was proven safe for NSCLC patients with CNS metastases [6]. One study of six patients, conducted in 2010 [6], found that three patients achieved stable disease and two achieved a partial response. Intracranial hemorrhage was observed in only one of three patients using bevacizumab and a concurrent anticoagulation agent.

EGFR-mutant lung cancer was first described in 2004 [7] and has since been characterized as a distinct subset of lung cancer. EGFR-TKIs such as gefitinib and erlotinib have yielded promising results against these cancers, and both agents have been shown to significantly increase survival. For example, one study of 17 patients with EGFR-mutated lung cancer with brain metastasis (exon 19 and 21 deletions) demonstrated that eight of nine patients who concurrently received high-dose erlotinib therapy and WBRT achieved a partial or complete response, compared with six of eight patients treated with erlotinib alone [8]. Patients in that study had a median time to progression of 11.7 months. Osimertinib, an irreversible EGFR-TKI currently in phase I trials is a promising candidate; patients who reached a treatment duration of 12 weeks exhibited symptomatic and radiologic improvements, and it is generally well tolerated [9].

More recently, ALK mutations have led to studies of crizotinib, an ALK-TKI that has yielded promising results [3]. However, pre-existing lesions tended to progress and/or new intracranial lesions tended to develop during crizotinib therapy. A recent phase II trial found that alectinib, a second-generation ALK-TKI, was efficacious in crizotinib-resistant cases [10].

## Conclusions

Although LC remains a complex disease with a grave prognosis, progress during the last decade has led to improved survival among affected patients. However, the treatment of LC requires an individualized approach. WBRT, systemic therapy, and targeted therapy have been evaluated as candidates for improving a patient's quality of life. The current research and treatments associated with genetic testing are promising, and the identification of mutations such as EGFR and ALK have facilitated the use of targeted therapies that have enhanced progression-free survival and overall survival. Although many of the treatments administered for LC remain palliative in nature, we have gained a better understanding of the obstacles that must be overcome to prolong survival and improve the quality of life of the affected patients.
